# Impact of COVID-19 pandemic on central-line–associated bloodstream infections during the early months of 2020, National Healthcare Safety Network

**DOI:** 10.1017/ice.2021.108

**Published:** 2021-03-15

**Authors:** Prachi R. Patel, Lindsey M. Weiner-Lastinger, Margaret A. Dudeck, Lucy V. Fike, David T. Kuhar, Jonathan R. Edwards, Daniel Pollock, Andrea Benin

**Affiliations:** 1 Division of Healthcare Quality Promotion, Centers for Disease Control and Prevention, Atlanta, Georgia; 2 CACI, Atlanta, Georgia

## Abstract

Data reported to the Centers for Disease Control and Prevention’s National Healthcare Safety Network (CDC NHSN) were analyzed to understand the potential impact of the COVID-19 pandemic on central-line–associated bloodstream infections (CLABSIs) in acute-care hospitals. Descriptive analysis of the standardized infection ratio (SIR) was conducted by location, location type, geographic area, and bed size.

The US Centers for Disease Control and Prevention’s (CDC) National Healthcare Safety Network (NHSN) is the nation’s surveillance system for healthcare-associated infections (HAIs). Hospitals and public health organizations track HAIs using the standardized infection ratio (SIR), and the Centers for Medicare and Medicaid Services (CMS) require submission of data on HAIs to the NHSN for payment programs such as the Hospital-Acquired Conditions Reporting Program (HACRP).^1^ From 2015 to 2019, there was a 31% decline in the national SIR for central-line–associated bloodstream infections (CLABSIs).^2^ However, in the face of the coronavirus disease 19 (COVID-19) pandemic, HAIs in hospitals may have increased.^3^,^4^ To understand the impact of the early months of the COVID-19 pandemic on CLABSIs nationally, SIRs for the second quarter of 2020 (2020 Q2: April, May, June) were compared to those from 2019 Q2.

## Methods

Reporting on CLABSIs to the NHSN should occur in any inpatient location where data on central lines can be collected, including intensive care units (ICUs), specialty care areas (SCAs), neonatal intensive care units (NICUs), and wards.^5^ In this analysis, we included data as of January 1, 2021, from acute-care hospitals (ACHs) for 2019 Q2 and 2020 Q2. Only locations that had continuous and consistent reporting, defined as ACHs reporting all 3 months of CLABSI data for the same location in both 2019 Q2 and 2020 Q2, were included. SIRs were calculated by dividing the number of observed infections by the predicted number determined from the logistic regression model generated from national data during a baseline period.^6^ A mid-*P* exact test was preformed to compare the 2020 Q2 SIRs to the baseline of 1 and to the 2019 Q2 SIRs. Device utilization ratios were calculated by dividing central-line days by patient days. Regions were defined by the US Department of Health and Human Services (HHS).^7^

Our analysis was restricted to the units included in the CMS HACRP and location types that had at least 20 reporting locations nationwide.^1^ Because CMS suspended the HACRP reporting requirement for HAIs during 2020 Q2, the number of reporting hospitals in 2020 Q2 was compared to 2019 Q2.

The percentage change in the SIR was calculated as follows: [(SIR for 2020 Q2 – SIR for 2019 Q2)/SIR for 2019 Q2 × 100]. A mid-*P* exact test was performed to estimate the 95% confidence intervals around SIR percentage change values. The percentile distribution of 2020 Q2 SIRs included those facilities that had a denominator of the SIR (ie, number predicted CLABSI) >1. Percentile distributions are shown for strata with >20 facility-level SIRs.

## Results

Our analysis included 13,136 inpatient units from 2,986 ACHs; 936 facilities had at least 1 predicted CLABSI and an SIR calculated. A 28% increase (95% CI, 20.0–33.6) was observed in the national SIR, from 0.68 in 2019 Q2 to 0.87 in 2020 Q2 (Table 1). Device utilization increased nationally from 0.21 in 2019 Q2 to 0.23 in 2020 Q2 (data not shown).

**Table 1.** Preliminary National Central-Line–Associated Bloodstream Infection (CLABSI) Standardized Infection Ratios (SIRs) in US Acute-Care Hospitals During the Early Months of the COVID-19 Pandemic, April 2020–June 2020

Characteristic	Reporting Hospitals in 2020 Q2^a^	Total Reporting Hospitals in 2019 Q2^b^	Observed CLABSI	Predicted CLABSI	Central- Line Days	2020 Q2 SIR	2019 Q2 SIR	SIR % Change^c^	95% Confidence Interval for the SIR % Change	Facility-Level Distribution of the Standardized Infection Ratio (SIR) for 2020 Q2^d^					
										10%	25%	50%	75%	90%	100%
All Hospitals	2,986	3,594	2,887	3,334.17	3,302,324	0.87^e^	0.68^e^	28.0^f^	(20.0 to 33.6)	0.00	0.00	0.68	1.17	1.96	6.72
**Location type**															
CC	2,513	3,070	1,911	1,836.02	1,707,981	1.04	0.75^e^	38.7^f^	(29.2 to 48.5)	0.00	0.00	0.78	1.51	2.31	6.49
CC_ONC	19	21	10	10.93	10,706	0.91	1.08	−15.7	(−63.2 to 89.6)	…	…	…	…	…	…
NICU	812	1,035	162	305.93	219,115	0.53^e^	0.65^e^	−18.5	(−33.4 to 0.5)	0.00	0.00	0.00	0.80	1.26	2.56
Ward^g^	2,852	3,478	804	1,181.29	1,364,522	0.68^e^	0.60^e^	13.3^f^	(2.3 to 23.9)	0.00	0.00	0.51	0.99	1.81	7.89
**Bed size**															
<100 beds	1,117	1,341	114	124.46	180,626	0.92	0.66^e^	39.4^f^	(4.2 to 82.7)	…	…	…	…	…	…
100–199 beds	774	921	418	429.09	520,542	0.97	0.74^e^	31.1^f^	(14.2 to 52.7)	0.00	0.00	0.77	1.56	2.42	6.24
200–299 beds	474	553	505	594.21	612,958	0.85	0.66^e^	28.8^f^	(13.0 to 46.7)	0.00	0.00	0.62	1.08	2.01	6.72
300– 499 beds	426	536	820	1,030.37	956,115	0.80^e^	0.62^e^	29.0^f^	(15.6 to 41.5)	0.00	0.00	0.64	1.20	1.99	4.76
500–699 beds	123	156	549	620.14	557,211	0.89	0.68^e^	30.9^f^	(15.7 to 48.3)	0.14	0.39	0.73	1.11	1.74	4.31
>700 beds	72	88	481	535.91	474,872	0.90	0.80^e^	12.5	(−0.8 to 27.9)	0.30	0.50	0.85	1.14	1.70	2.17
**HHS Region**															
1 - Upper Northeast	116	147	137	128.45	133,833	1.07	0.74^e^	44.6^f^	(11.3 to 88.4)	0.00	0.00	0.92	1.19	2.72	4.48
2 - Middle Northeast	131	254	111	159.39	162,136	0.70^e^	0.67^e^	4.5	(−20.0 to 36.6)	0.00	0.00	0.42	1.31	1.86	3.46
3 - Lower Northeast	286	336	357	379.91	368,526	0.94	0.70^e^	34.3^f^	(14.4 to 56.3)	0.00	0.00	0.70	1.14	1.99	3.83
4 - Southeast	694	786	660	802.99	786,484	0.82^e^	0.72^e^	13.9^f^	(2.0 to 26.9)	0.00	0.12	0.65	1.07	1.89	6.72
5 - Great Lakes	460	576	498	525.58	514,927	0.95	0.71^e^	33.8^f^	(16.7 to 51.7)	0.00	0.00	0.67	1.40	1.97	4.82
6 - South Central	494	597	366	442.18	442,148	0.83^e^	0.76^e^	9.2	(−6.2 to 25.4)	0.00	0.31	0.71	1.16	1.83	2.90
7 - Middle Plains	163	188	134	160.40	157,863	0.84^e^	0.68^e^	23.5	(−4.1 to 56.1)	0.00	0.00	0.75	1.11	1.91	2.62
8 - Northern Plains	135	143	107	107.02	106,249	1.00	0.62^e^	61.3^f^	(19.9 to 121.2)	0.00	0.45	0.81	1.54	2.18	2.83
9 - West	400	454	434	508.19	505,247	0.85^e^	0.57^e^	49.1^f^	(30.2 to 74.7)	0.00	0.00	0.63	1.20	2.28	4.64
10 - Northwest	105	112	82	119.25	123,656	0.69^e^	0.45^e^	53.3^f^	(10.3 to 114.6)	0.00	0.00	0.61	1.17	1.90	2.70

Note. CC, critical care; CC_ONC, oncology critical care; NICU, neonatal intensive care units; HHS, Department of Health and Human Services.

a Includes hospitals reporting three months of complete CLABSI surveillance data for the same location in both 2019 Q2 and 2020 Q2.

b Includes all hospitals reporting 3 months of complete CLABSI surveillance data for 2019 Q2 (will be greater than consistent and continuous reporters).

c Calculated as follows: [(SIR for 2020 Q2 – SIR for 2019 Q2)/SIR for 2019 Q2 × 100].

d If there were <20 SIRs nationally, the distribution is displayed as missing.

e Significantly different from 1; *P* < .05.

f Significant difference between 2020 Q2 and 2019 Q2 SIRs; *P* < .05.

g Includes the following location types: medical ward, medical surgical ward, pediatric medical surgical ward, pediatric medical ward, surgical ward, and pediatric surgical ward.

Critical care units had the greatest percentage increase (39%) in SIR, from 0.75 in 2019 to 1.04 in 2020. Ward locations experienced the second highest increase (13%). Critical care locations had the highest number of CLABSIs in 2020 Q2, with 1,911 events. Hospitals in all bed-size categories exhibited an increase in SIR.

In 2020 Q2, reporting of CLABSI surveillance dropped by 17% nationally, in contrast with 2019 Q2. The greatest decrease in reporting (48%) occurred in the Middle Northeast. Regional analysis showed significant percentage changes in the SIR from 2019 to 2020 in 7 regions: Upper Northeast, Lower Northeast, Southeast, Great Lakes, Northern Plains, West, and Northwest. The highest regional 2020 Q2 SIR was 1.07 and occurred in the Upper Northeast, representing a 45% increase compared to 2019 Q2.

Evaluating by ward type, pediatric medical-surgical wards contributed 4% of the national central-line days from ward locations in 2020 Q2 and had the greatest change in their SIR (118% increase) (Table 2). Statistically significant increases in the SIR also occurred in medical critical care (60%), medical-surgical critical care (59%), and neurosurgical critical care (108%). In addition, the device utilization ratio increased in pediatric medical-surgical wards from 0.14 (2019) to 0.18 (2020) (data not shown).

**Table 2.** Preliminary National Central-Line–Associated Bloodstream Infection (CLABSI) Standardized Infection Ratios (SIRs) During the Early Months of the COVID-19 Pandemic, by Location Type, April 2020–June 2020

Location Type	No. of Locations	Observed CLABSI	Predicted CLABSI	Central-Line Days	2020 Q2 SIR	2019 Q2 SIR	SIR % Change^a^	95% Confidence Interval for the SIR % Change	Location Type distribution of the Standardized Infection Ratio (SIR) for 2020 Q2^b^					
									10%	25%	50%	75%	90%	100%
**Critical care locations**														
Burn critical care	51	29	52.58	15,819	0.55^c^	0.44^c^	25.3	(−28.1 to 120.6)	0.00	0.00	0.10	0.96	1.42	2.44
Medical cardiac critical care	211	91	87.17	81,439	1.04	1.06	−1.1	(−26.0 to 32.3)	…	…	…	…	…	…
Surgical cardiothoracic critical care	341	116	193.66	180,522	0.60^c^	0.64^c^	−6.3	(−26.9 to 20.0)	0.00	0.00	0.57	0.96	1.60	2.92
Pediatric surgical cardiothoracic critical care	44	40	68.48	43,248	0.58^c^	0.50^c^	16.0	(−26.7 to 84.20)	0.00	0.00	0.51	0.88	1.25	2.10
Medical critical care	584	397	274.19	263,176	1.45^c^	0.91	59.6^d^	(35.9 to 87.7)	0.00	0.00	1.21	2.35	2.78	7.39
Medical-surgical critical care	2,076	885	727.37	774,905	1.22^c^	0.77^c^	58.9^d^	(42.6 to 77.3)	0.00	0.00	0.93	1.80	3.35	5.21
Pediatric medical-surgical critical care	237	76	106.36	70,845	0.71^c^	0.66^c^	8.3	(−20.8 to 47.7)	0.00	0.19	0.78	1.06	1.87	2.96
Pediatric medical critical care	27	8	5.73	3,944	1.40	1.50	−6.9	(−66.3 to 156.8)	…	…	…	…	…	…
Neurologic critical care	73	21	28.58	26,628	0.73	0.73	0.4	(−46.4 to 88.7)	…	…	…	…	…	…
Neurosurgical critical care	157	62	67.29	61,448	0.92	0.44^c^	107.9^d^	(34.5 to 226.8)	…	…	…	…	…	…
Surgical critical care	260	136	135.29	125,839	1.01	0.93	8.6	(−14.8 to 38.3)	0.00	0.00	0.81	1.20	1.96	3.56
Trauma critical care	126	48	85.99	57,473	0.56^c^	0.56^c^	−0.5	(33.2 to 48.1)	0.00	0.00	0.35	0.93	1.74	2.84
**NICU**														
Neonatal critical care (level II/III)	514	68	118.07	84,535	0.58^c^	0.66^c^	−13.0	(−37.1 to 20.0)	0.00	0.00	0.00	0.94	1.45	2.56
Neonatal critical care (level III)	330	94	187.39	134,080	0.50^c^	0.63^c^	−20.9	(−39.6 to 3.3)	0.00	0.00	0.00	0.80	1.13	1.96
**Wards**														
Medical ward	2134	251	381.28	435,198	0.66^c^	0.64^c^	2.8	(−13.1 to 21.4)	…	…	…	…	…	…
Medical-surgical ward	3,985	346	512.83	619,386	0.67^c^	0.59^c^	14.3	(−1.3 to 32.5)	…	…	…	…	…	…
Pediatric medical-surgical ward	564	49	54.65	53,821	0.90	0.41^c^	117.6^d^	(37.1 to 249.5)	…	…	…	…	…	…
Pediatric medical ward	180	19	22.89	21,839	0.83	0.75	10.2	(−40.4 to 101.9)	…	…	…	…	…	…
Surgical ward	1,168	134	202.84	228,044	0.66^c^	0.60^c^	10.6	(−12.6 to 40.1)	…	…	…	…	…	…
Pediatric surgical ward	30	5	6.80	6,234	0.74	0.76	−3.4	(−71.3 to 198.5)	…	…	…	…	…	…

Note. NICU, neonatal intensive care units.

a Calculated as follows: [(SIR for 2020 Q2 – SIR for 2019 Q2)/SIR for 2019 Q2 × 100].

b If there were <20 SIRs nationally, the distribution is displayed as missing.

c Significantly different from 1; *P* < .05.

d Significant difference between 2020 Q2 and 2019 Q2 SIRs; *P* < .05.

## Discussion

The national SIR for CLABSIs increased significantly by 28% in 2020 Q2 versus 2019 Q2. During that same time, hospitals were faced with managing the emerging pandemic of COVID-19, which may have played a role in the increase. Infection control practices changed in many healthcare settings during the pandemic to accommodate increasing numbers of patients and to mitigate shortages of personal protective equipment, supplies, and staffing.^4^ Reducing the frequency of contacts with patients and of maintenance activities for central venous catheters (eg, chlorhexidine bathing, scrubbing the hub, site examinations) as well as alterations to processes of care (eg, risking disrupting catheter dressings when placing patients in a prone position) all have the potential to contribute to an increase in CLABSIs.^4^

Consistent with the concern that high-acuity care for patients with COVID-19 posed heightened challenges for preventing device-associated infections; CLABSIs in critical care locations occurred relatively frequently in the 2020 data. The number of CLABSIs in those locations exceeded CLABSIs in ward locations by 1,100 events for the quarter. In prior years, the number of CLABSIs identified in ward locations would typically exceed the number reported from ICUs.^2^ NHSN data do not enumerate the specific type of ICU location of patients with COVID-19, but among all ICUs, increases CLABSIs were highest in the medical-surgical critical care units. The significant 59% increase in the 2020 SIR highlights the likely burden that was placed on these units.

The reporting of data on CLABSIs decreased across all regions, with 609 fewer hospitals reporting in 2020 Q2. This drop in reporting may have affected the regional-level analyses because alterations in reporting may have occurred disproportionally in regions with more COVID-19 patients. In particular, even though New York and New Jersey experienced increased hospitalizations during this period, the Middle Northeast region did not demonstrate a significant increase in SIR, and this region had the largest decline in reporting of CLABSIs by 48%.^8^ In contrast, the Southeast region had only a 12% drop in reporting of data on CLABSIs to NHSN, and the analyses were able to discern an increase in CLABSI SIR against the backdrop of an increase in hospitalizations due to COVID-19 during June 2020.^8^

The analysis had several limitations. Results were restricted to locations for which data were consistently reported in both 2019 and 2020 Q2. New locations that may have been created in 2020 in response to patient surge due to the pandemic were not included because they were not present in 2019 for comparison analysis. Restricting the analyses to those units required by CMS HACRP may have excluded other units that were used by hospitals for COVID-19 patients (eg, pulmonary wards).

These findings highlight a substantial increase in CLABSIs in hospitals throughout the United States coinciding with the COVID-19 pandemic. The results of this analysis can be used to understand the increase in HAI burden being placed on the nation’s healthcare system and to prioritize ongoing efforts to prevent infections and to drive patient safety.
